# The onset of nausea and vomiting of pregnancy: a prospective cohort study

**DOI:** 10.1186/s12884-020-03478-7

**Published:** 2021-01-06

**Authors:** Roger Gadsby, Diana Ivanova, Emma Trevelyan, Jane L. Hutton, Sarah Johnson

**Affiliations:** 1 Department of Statistics, University of Warwick, Coventry, CV5 6AB UK; 2 Warwick Medical School, University of Warwick, Coventry, CV5 6AB UK; 3 SPD Development Company Limited, Stannard Way, Bedford, MK44 3UP UK

**Keywords:** Pregnancy, Nausea, Vomiting, Onset, Last menstrual period, Luteinising hormone (LH) surge

## Abstract

**Background:**

Nausea and vomiting are experienced by most women during pregnancy. The onset is usually related to Last Menstrual Period (LMP) the date of which is often unreliable. This study describes the time to onset of nausea and vomiting symptoms from date of ovulation and compares this to date of last menstrual period

**Methods:**

Prospective cohort of women seeking to become pregnant, recruited from 12 May 2014 to 25 November 2016, in the United Kingdom. Daily diaries of nausea and vomiting were kept by 256 women who were trying to conceive. The main outcome measure is the number of days from last menstrual period (LMP) or luteinising hormone surge until onset of nausea or vomiting.

**Results:**

Almost all women (88%) had Human Chorionic Gonadotrophin rise within 8 to 10 days of ovulation; the equivalent interval from LMP was 20 to 30 days. Many (67%) women experience symptoms within 11 to 20 days of ovulation.

**Conclusions:**

Onset of nausea and vomiting occurs earlier than previously reported and there is a narrow window for onset of symptoms. This indicates that its etiology is associated with a specific developmental stage at the foetal-maternal interface.

**Trial registration:**

NCT01577147. Date of registration 13 April 2012

**Supplementary Information:**

The online version contains supplementary material available at (doi:10.1186/s12884-020-03478-7).

## Background

Nausea and vomiting of pregnancy (NVP) has been recognised as a feature of early pregnancy for well over 2000 years. Hippocrates who lived from 460 to 370 BC wrote “when a woman who is suffering from neither rigours nor fever, develops amenorrhea and is liable to nausea, she is pregnant” [1]. Different national guidelines recommend different approaches to the management of NVP, with a wide range of recommendations [2].

The precise date and the characteristics of its onset have been more difficult to define. Several papers that describe onset [3, 4] have collected data retrospectively by asking women to describe their onset when they attend their first ante-natal clinic often at around 12 weeks from last menstrual period (LMP). Such retrospective data may be particularly unreliable if symptoms are mild and have subsided by the time of data collection. In the Canadian study, 74% of women reported nausea [4]. A study describing the onset and natural history of NVP that asked women to present when they first thought they were pregnant so as to enter the study as early as possible in order to collect symptoms prospectively, reported entry at a median of day 57 from LMP. In that study 58 (16%) of women out of a study population of 363 reported symptoms before entry, some of them up to 14 days before entry [5]. Using LMP to date onset of pregnancy nausea and vomiting may also be problematic. Some studies have shown that only 32% of women were certain of the date of their LMP [6]. A higher incidence of round number preferences were recorded when women were asked the date of their LMP with the 15th of the month given 2.5 times more often than expected [7].

A much more accurate way of dating a pregnancy is from using the date of ovulation which can be determined by measuring the surge in luteinising hormone (LH) that stimulates ovulation [8]. The cause of NVP is still largely unknown, but is thought to be multifactorial, with a genetic element in its aetiology [9]. A psychiatric aetiology has been proposed in the past but, although this has been disproven [10], sufferers feel that this remains an inhibitor to the empathy and care the condition deserves [10]. Women who have experienced severe NVP are very keen to stress the biological nature of the condition [11]. Evidence that there is a very close association between the onset of NVP symptoms and the onset of pregnancy, as measured from the date of ovulation, would provide further evidence for a biological aetiology.

In this paper we study the onset of NVP in relation to the date of ovulation and LMP

## Methods

A cohort study conducted by SPD Development Company Limited (SPD) was originally designed to investigate hormone levels in early pregnancy. It was extended to include the study of pregnancy sickness and vomiting; the methodology has described previously [12]. In brief, the study was registered (NCT01577147) and ran from 15 May 2014 to 17 February 2017. Volunteers were recruited preconception, and all gave written consent for the home-based study. Inclusion criteria were: at least 18 years old and seeking to become pregnant and has regular menstrual periods. Exclusion criteria were: medical conditions that contra-indicate pregnancy, known infertility or having treatment for infertility or women who were currently pregnant.

Volunteer demographics, menstrual cycle information and previous pregnancy history were collected at admission. From day 1 of their next menstrual cycle, volunteers collected a daily early morning urine sample and completed a daily diary. Lutenising hormone (LH) was measured (AutoDELFIA, Perkin Elmer) in order to determine the day of LH surge, which precedes ovulation by approximately 1 day. Human Chorionic Gonadotrophin (hCG) was measured (AutoDELFIA, Perkin Elmer) in late luteal phase and throughout early pregnancy, in order to identify viable pregnancy and early pregnancy loss.

Home pregnancy tests were provided to volunteers. When a volunteer reported pregnancy to the study site, she was instructed to record each hour of nausea and vomiting symptoms in a diary. The questionnaire was adapted from the PUQE score system for recording nausea and vomiting to allow more details of times (Supple-mentary material 1: Diary). The instructions were to start recording on day 30 following LMP (date of last menstrual period), and stop at day 60 from LMP. Hence participants started the diaries on different days of their pregnancy. Woman who did not achieve pregnancy were instructed to collect urine samples and complete symptom diaries only up to day 7 of her next cycle.

**Statistical methods**

The anonymised dataset was transferred to the Department of Statistics at the University of Warwick and examined by Master’s students using R Studio statistical Software. Standard summary statistics, linear regression and significance tests were used. For the table, age and hours per day of symptoms were split approximately into thirds, and body mass index (BMI, kg/m^2^) into normal, overweight and obese categories. For investigation of time to onset of symptoms by age and BMI, the variables were continuous. A NVP score was calculated as the average daily hours of nausea and vomiting based on the time period the women reported, using available data. A high level summary of symptom severity is given; results for diurnal variation are given elsewhere [12].

## Results

### Study population

Initially, 1443 women who were planning to become pregnant were recruited into the study. 1073 of those women did not achieve pregnancy in a one-month period, 17 women had a miscarriage and 65 women had early losses. Out of the remaining 288, who successfully conceived, 32 dropped out of the study. Hence, the study population includes 256 women. Only 866 samples of the expected 16830 urine samples were missing from the daily sample collections, providing 95% coverage.

Many, 60%, of the women had previously had live births, mainly one child (Table 1, Total column). The mean age was 30.4 years, the mean age of mothers in England and Wales in 2016 [13]; age ranged from 18 to 43. The majority (54%) of younger women (<28) in the study had no children, and half of women over 32 had one child. Most (95%) of the women were white and were European. The mean BMI was 27.0 kg/m ^2^, standard deviation 6.1 kg/m ^2^. Half (53%) the women were overweight or obese, and 9% were underweight; one BMI was missing. Almost half (43%) of the population had previously suffered at least one miscarriage, with more miscarriages associated with more live births. Most participants (75%) had an education level of A-levels or higher, and more of these women (43%) had no previous live births, although those with degrees were older than those without A-levels. Before conceiving during this study, volunteers had been trying for a mean of 8.42 months, median of 5 months.

**Table 1:** Characteristics of 256 women in nausea and vomiting diary study

Characteristic	Average hours per day Nausea and Vomiting			Totals
	<1,5 hours/day	(1.5,4] hours/day	≥4 hours/day	
All women	56 (22%)	108 (42%)	92 (36%)	256 (100%)
Age				
18-28	20 (24%)	40 (48%)	24 (29%)	84 (33%)
29-32	22 (23%)	44 (45%)	31 (32%)	97 (38%)
33-43	14 (19%)	24 (32%)	37 (49%)	75 (29%)
BMI				
≤25kg/m^2^	32 (27%)	53 (45%)	34 (29%)	119 (46%)
(25,30] kg/m^2^	18 (24%)	30 (32%)	28 (44%)	63 (25%)
>30 kg/m^2^	8 (11%)	34 (47%)	30 (42%)	72 (28%)
Smoking				
Current or ex	37 (24%)	54 (35%)	42 (42%)	100 (39%)
Never	19 (19%)	54 (54%)	27 (47%)	156 (61%)
Previous live births				
0	28 (27%)	52 (50%)	23 (22%)	100 (40%)
1	21 (20%)	39 (37%)	45 (43%)	105 (41%)
≥2	7 (15%)	17 (35%)	24 (50%)	48 (19%)
Miscarriages				
0	35 (24%)	60 (41%)	50 (34%)	145 (57%)
1	12 (18%)	35 (51%)	21 (31%)	68 (27%)
≥2	9 (21%)	13 (30%)	21 (49%)	43 (17%)
Education level				
Below A-level	11 (17%)	35 (56%)	17 (27%)	63 (25%)
A-levels	16 (19%)	32 (38%)	36 (43%)	84 (33%)
Degree level	29 (27%)	41 (38%)	39 (36%)	109 (43%)

### Daily diaries

Only 10 women had any missing daily symptom diaries; 9 missed a single day and one missed three days. As this is negligible, the overall NVP scores for each women used available data. 61% (n = 148) of the volunteers with NVP started filling in their daily diaries before they had their first symptoms. The remaining 93 participants first filled in their daily diaries on the first day they experienced symptoms. Their mean onset was a week earlier than those who started diaries before first symptoms, so there is no reason to believe symptoms occured before the first recorded day.

Most women, 94.1% (n= 241) had some symptoms of NVP. In particular, 35% (n = 89) of the women experienced just nausea, 59% (n= 150) suffered from both nausea and vomiting. Two volunteers reported to have had only vomiting, however the intensity was respectively 1 hour and 4 hours of sickness (vomiting) throughout their whole pregnancies. Women with more than one previous live births experience significantly longer average hours of symptoms per day, median 3.9 hours.

### LMP vs. ovulation

**Fig. 1:**
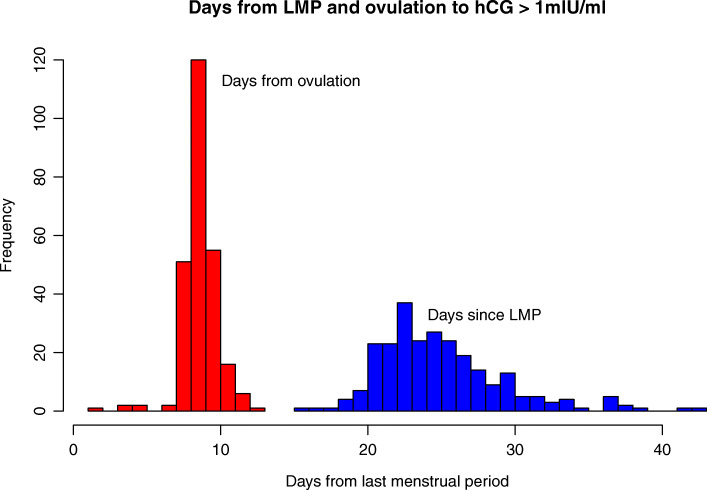
Days from LMP and from Ovulation day to hCG >1mlU/ml (256 women)

By convention and in all relevant to NVP literature, LMP is considered as the beginning of pregnancy. Nevertheless, this is somewhat inaccurate as for women with monthly cycles of different lengths, the time between LMP and ovulation and hence between LMP and fertility or impregnation varies. Nevertheless, as the ovulation day can be calculated by the LH surge, it was included in this data set. The inclusion of both of these dates in the data set allows for a comparison of their accuracy as a measure of the start of pregnancy. As the concentration of hCG is an ideal marker of pregnancy [8], comparing the dates of LMP and ovulation to the first date when the pregnancy hormone hCG first reached a concentration of 1 mIU/ml (the hCG rise) can be expected to give an accurate result of the comparison. Figure 1 compares the duration to start of pregnancy. Almost all women (88%) had hCG rise within 8 to 10 days of ovulation. Only 3% had intervals less than 8 days, and 9% more than 10 days. The time between LMP and the hCG rise varies significantly more, with 88% within 20 days to 30 days (11 days as opposed to 3 days), and 9% 31 to 60 days (Table 2). This demonstrates that the day of ovulation, calculated by the LH surge, is a much more precise indicator of the start of pregnancy than the first day of the last menstrual period.

**Table 2:** Times to hCG rise (256 women) and to onset of symptoms (241 women)

Time	Summary statistics in days				
	Minimum	Lower	Median	Upper	Maximum
		quartile		quartile	
LMP to hCG >1mlU/ml	15	23	25	28	60
Ovulation to hCG >1mlU/ml	1	9	9	10	13
LMP to symptom onset	21	28	32	40	66
Ovulation to symptom onset	8	13	16	22	45

### Onset of symptoms

**Fig. 2:**
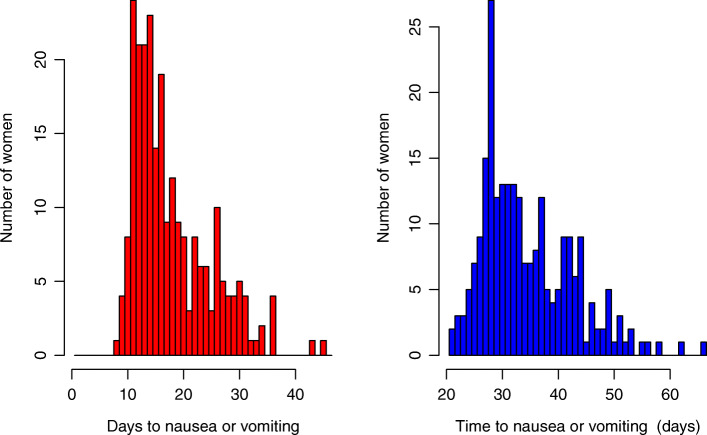
Distribution of onset of symptoms from LMP and from Ovulation (214 women)

**Fig. 3:**
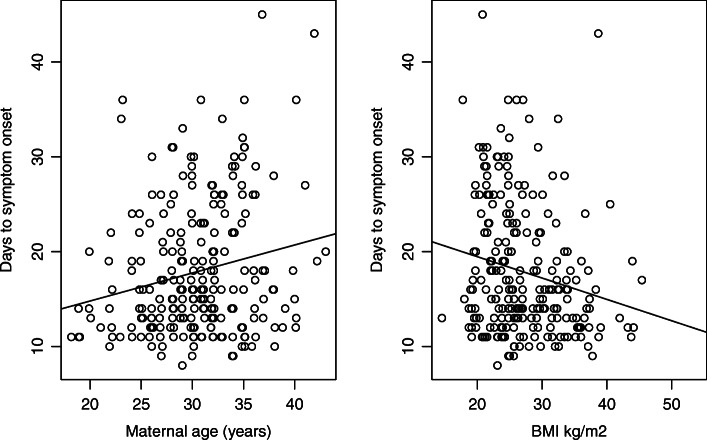
Onset of symptoms from ovulation: association with age and body mass index (BMI), 214 women

From ovulation, symptoms start approximately two weeks earlier, and are more concentrated than from LMP (Fig. 2). Two-thirds of women have onset within 11 to 20 days from ovulation day, and only 5% have earlier onset. The median from ovulation to symptom onset is 16 days and from LMP is 32 days. For onset from LMP, 67% have onset within 26 to day 40 (Fig. 2), with highest frequency at 28 days after LMP. Again, precision is greater when measuring pregnancy from ovulation (Table 2). Symptoms start slightly earlier for women with higher BMI values (Fig. 3), by about one day for an increase of 5 kg/m^2^ (regression coefficient -0.225, standard error 0.075). Predicted onset is day 20 for a woman with BMI 18 kg/m^2^ and day 16 for a woman with BMI 35 kg/m ^2^. Symptoms start slightly later with increasing age (Fig. 3), by almost one day for an increase of 3 years (regression coefficient 0.295, standard error 0.094). Predicted onset is day 14 for an 18 year old, and day 21 for a 41 year old woman.

Symptoms disappeared by week 7 from LMP for 20 (8%) of the volunteers, 11 of whom never vomited and 16 of whom had symptoms less than 2 hours per day. Furthermore, out of the 25 women whose symptoms ended in week 8 of their pregnancies, 15 had symptoms less than 2 hours per day. These 32-35 women (12-14% of the study population) with early or mild symptoms might have not recorded them if they were first asked about their symptoms at a later stage of their pregnancy.

### Severity of symptoms

Table (1) provides a high level summary of symptom severity considered as average hours per day. A fifth (22%) of women had minimal nausea and vomiting, with an average of less than an hour per day; 36% averaged more than 4 hours per day. Smokers and those with at least two previous live births tended to have symptoms for longer each day. There is no simple association with miscarriages, and a slight suggestion of lower symptom rates for women with degree level education.

## Discussion

### Main findings

This paper describes for the first time the onset of symptoms of nausea and vomiting in pregnancy from the day of ovulation. The vast majority of women (94%) experienced symptoms which started within a clear, narrow time window from ovulation. In particular, symptoms began between days 11 and 20 for 67% (n = 160) of the 241 par- ticipants with NVP. When LMP is used to date the onset of pregnancy, as has been done in previously published studies, the equivalent range is days 26 to 40. Onset was later with increasing age, and slightly earlier with increasing BMI. The close connection between onset of NVP symptoms and date of ovulation reinforces the theory that the aetiology of the condition is biological, rather than psychiatric, and is based on the emerging physiology at the foetal-maternal interface.

### Interpretation

Onset of symptoms of nausea or vomiting was earlier than reported in other articles: median from LMP at day 32, compared to day 57, 8 weeks [5] or 5 weeks [4]. A possible reason for this discrepancy is that the volunteers in this study were all women who have been trying to get pregnant for a while and would be awaiting early mild NVP symptoms as one of the first signs they were pregnant. Moreover, in all of the studies mentioned above the initial interview or survey was at least 8-9 weeks after onset. In this setting some of the participants who had experienced brief symptoms in the beginning of their pregnancies might have forgotten about them when they had to report their experiences of symptoms retrospectively, [14].

The prevalence of NVP symptoms in this study, at 94%, is higher than that reported in other studies, which have reported a prevalence of NVP symptoms in 50-80% of pregnant women [4, 5]. In a literature review that included data from 26 published papers, of 39710 pregnancies, the percentage of pregnant women experiencing NVP symptoms was 73.4% [15]. A meta-analysis of 59 studies of NVP (93,753 women) reported a median rate of 69%, range 35% to 91%; rates for East Asian countries were between 75% and 91% [16]. Prevalence estimates from clinical records of NVP are much lower 9.1% [17]. Again, one reason may be that the women were actively trying to conceive and this might have made them more inclined to notice details related to their pregnancies such as mild symptoms. Another reason could be that most previous studies of NVP onset recorded at least some of their data retrospectively. Mild early symptoms of NVP may get overlooked and forgotten, especially if women are being asked to remember them a number of weeks later. Detailed results for factors affecting symptoms are a separate article.

This hypothesis was confirmed in this study in that 13% (n=32) of women who had very early symptoms up to week 7 or mild symptoms, lost them by day 56 (week 8) from LMP, the date at which many previous studies began their prospective recording of NVP symptoms. The mean day of onset of NVP symptoms in this study is day 18 from ovulation, while from LMP it is day 34. This date from LMP is slightly earlier than 39 days [5] or 40 days (5.7 weeks) [4] reported previously. In a review of onset of NVP symptoms from LMP, data from 7 papers with a pooled population of 2092 women, had mean day of onset from LMP as day 39 [15].

### Strengths and limitations

The strengths of our study are that it is based on a sizable cohort of women who are broadly representative of women planning to become pregnant in the UK. The data on NVP symptoms was collected prospectively from the onset of pregnancy and we can pinpoint the day of ovulation through the measurement of the LH surge. As the mean onset of NVP for those who started their pregnancy diaries on the first day of symptoms was a week earlier than those who started diaries before first symptoms, it is likely that symptoms did not occur before the first recorded day. Some weaknesses of the study are that the dataset under-represents women from lower socio-economic (educational) backgrounds, and black and minority ethnic backgrounds. Unplanned pregancies were not included, but there is no evidence of difference in NVP for planned or unplanned pregnancies. East Asian countries report higher prevalence of NVP [16] but onset is not addressed. These results could be generalisable across education backgrounds for developed countries. Women in the study stopped recording NVP symptoms at around week 9 of pregnancy when several of them were still experiencing symptoms so that this dataset cannot be used to reliably describe NVP symptom cessation.

## Conclusion

Onset of NVP occurs earlier than previously reported and there is a narrow window for onset of symptoms from ovulation. This indicates that its etiology is associated with a specific developmental stage. Further study of the association between levels of hCG in urine and NVP symptoms could help to indicate more effective treatment.

## Supplementary Information

**Additional file 1** Description: Diary given to volunteers to complete from day 30 after LMP, with confidential data management elements removed.

## Data Availability

The datasets analysed during the current study are available from the corresponding author on reasonable request.

## Abbreviations

BMI: Body mass index
hCG: Human chorionic gonadotrophin
LH: Luteinising hormone
LMP: Last menstrual period
NVP: Nausea and vomiting of pregnancy
SPD: SPD development company limited
