# Supplementary material for: The onset of nausea and vomiting of pregnancy: a prospective cohort study
Source: BMC Pregnancy Childbirth. 2021 Jan 6;21:10. doi: 10.1186/s12884-020-03478-7 (PMC7786982; doi:10.1186/s12884-020-03478-7)
Supplement: Supplementary file 1 — Additional file 1 Description: Diary given to volunteers to complete from day 30 after LMP, with confidential data management elements removed. [file 12884_2020_3478_MOESM1_ESM.pdf]

Weekly Diary of Pregnancy Sickness

Instructions : For each day, please enter the date and then shade in the circles for the hours in which nausea and/or vomiting (including forcible retching) occur. At the end of each day please enter the actual number of occurrences of vomiting or retching for that day in the 'totals' box.
If no nausea or vomiting occurs, then simply enter a date, leave all circles unshaded and put '0' in the totals box.
Please answer questions 1 and 2 at the end of every week.

Date (Enter as : dd-mmm-yy)	Hour of day	Morning												Afternoon												No. of times vomited or retched per day
		00	01	02	03	04	05	06	07	08	09	10	11	12	13	14	15	16	17	18	19	20	21	22	23	
☐☐ - ☐☐☐ - ☐☐	Nausea	○	○	○	○	○	○	○	○	○	○	○	○	○	○	○	○	○	○	○	○	○	○	○	○	☐☐
	Sickness	○	○	○	○	○	○	○	○	○	○	○	○	○	○	○	○	○	○	○	○	○	○	○	○	
☐☐ - ☐☐☐ - ☐☐	Nausea	○	○	○	○	○	○	○	○	○	○	○	○	○	○	○	○	○	○	○	○	○	○	○	○	☐☐
	Sickness	○	○	○	○	○	○	○	○	○	○	○	○	○	○	○	○	○	○	○	○	○	○	○	○	
☐☐ - ☐☐☐ - ☐☐	Nausea	○	○	○	○	○	○	○	○	○	○	○	○	○	○	○	○	○	○	○	○	○	○	○	○	☐☐
	Sickness	○	○	○	○	○	○	○	○	○	○	○	○	○	○	○	○	○	○	○	○	○	○	○	○	
☐☐ - ☐☐☐ - ☐☐	Nausea	○	○	○	○	○	○	○	○	○	○	○	○	○	○	○	○	○	○	○	○	○	○	○	○	☐☐
	Sickness	○	○	○	○	○	○	○	○	○	○	○	○	○	○	○	○	○	○	○	○	○	○	○	○	
☐☐ - ☐☐☐ - ☐☐	Nausea	○	○	○	○	○	○	○	○	○	○	○	○	○	○	○	○	○	○	○	○	○	○	○	○	☐☐
	Sickness	○	○	○	○	○	○	○	○	○	○	○	○	○	○	○	○	○	○	○	○	○	○	○	○	
☐☐ - ☐☐☐ - ☐☐	Nausea	○	○	○	○	○	○	○	○	○	○	○	○	○	○	○	○	○	○	○	○	○	○	○	○	☐☐
	Sickness	○	○	○	○	○	○	○	○	○	○	○	○	○	○	○	○	○	○	○	○	○	○	○	○	
☐☐ - ☐☐☐ - ☐☐	Nausea	○	○	○	○	○	○	○	○	○	○	○	○	○	○	○	○	○	○	○	○	○	○	○	○	☐☐
	Sickness	○	○	○	○	○	○	○	○	○	○	○	○	○	○	○	○	○	○	○	○	○	○	○	○	

Q1. Does eating make your symptoms ... (put an 'X' in **one** box only)

☐ Better ☐ Worse ☐ No difference

Q2. On a scale of 0 - 10 (where 0 is "Worst possible" and 10 is "The best you felt before pregnancy"), how have your sickness symptoms been this week?
(put an 'X' in **one** box only)

0	1	2	3	4	5	6	7	8	9	10
☐	☐	☐	☐	☐	☐	☐	☐	☐	☐	☐
